# Birth weight was associated with maternal exposure to intimate partner violence during pregnancy in southern Ethiopia: A prospective cohort study

**DOI:** 10.3389/fpubh.2022.960443

**Published:** 2022-11-02

**Authors:** Sewhareg Belay, Ayalew Astatkie, Sven Gudmund Hinderaker

**Affiliations:** ^1^School of Public Health, College of Medicine and Health Sciences, Hawassa University, Hawassa, Ethiopia; ^2^Centre for International Health, University of Bergen, Bergen, Norway

**Keywords:** birth weight, newborn, intimate partner violence, pregnancy, Ethiopia

## Abstract

**Introduction:**

Birth weight is defined as the first weight of the newborn, ideally measured soon after birth. A recent Ethiopian survey estimated that 48% of births took place in health facilities. Data for women exposed to intimate partner violence (IPV) may be lacking in official statistics because these women may prefer to deliver at home, where data from non-institutional births, including reporting of birth weights, are not routinely recorded.

**Objective:**

The aim of this study was to investigate the association between maternal exposure to IPV during pregnancy and birth weight in a community in the Wondo Genet district of southern Ethiopia.

**Methods:**

We carried out a community-based prospective cohort study from February to December 2017. We followed up with 505 pregnant women and their newborns until after delivery. An interview about partner violence was done during pregnancy at home when enrolled. Field assistants who visited the homes measured the birth weight of each baby in grams. Twins and late birth weight measurements were excluded. Factors associated with birth weight were assessed by multiple linear regression.

**Results:**

Birth weight was assessed within 48 h for 477 (94.5%) newborns and between 48 and 72 h for an additional 28 (5.5%). There were 365 (72.3%) institutional deliveries. In an adjusted regression analysis (IPV adjusted for socio-economic status), birth weight was 203 g lower (B −203 95% CI −320 to −87) among newborns of women exposed to IPV than among the unexposed. Birth weight was also lower in girls than in boys, in newborns delivered at home rather than in a health facility, and in babies with a younger gestational age.

**Conclusion:**

Maternal exposure to IPV during pregnancy was associated with lower baby birth weights. Antenatal clinics should consider routinely identifying IPV-exposed women, and identifying babies with lower birth weights at home is an important indicator.

## Introduction

The birth weight of a fetus is an indicator of intrauterine growth and is the result of interactions between genetic, environmental, and social factors (1). Birth weight is defined as the first weight of the fetus or the newborn obtained after birth, ideally measured as soon as possible after birth to avoid the normal slight loss of bodyweight post-partum (2). The World Health Organization defines low birth weight (LBW) as a birth weight of <2,500 grams (5.5 pounds), irrespective of gestational age (2). LBW is either the result of preterm birth or restricted fetal growth (3). In 2015, 15% of babies globally (20.5 million) had LBW. About 25% of all babies with low birth weight are found in Africa, with the highest proportion being located in Eastern and Western Africa (4). Lower birth weight is also associated with increased perinatal and infant mortality (5). Babies with low birth weight have a higher risk of wasting, stunting, and being underweight during childhood (6). They also have increased risks later in life for being overweight, obese, and for developing several diseases, including diabetes mellitus (7). In 2012, the World Health Assembly published plans aiming to reduce the low birth rate by 30% before 2025 (8).

Does intimate partner violence (IPV) during pregnancy harm the baby? A systematic review and meta-analysis of studies conducted mainly in high-income countries showed that women who experienced IPV during pregnancy had an 18% higher chance of having a baby with LBW (9). A large study in Vietnam found that household economy and the educational status of the woman were factors associated with birth weight (10). Moreover, LBW was more common among neonates of mothers with prior experience of stillbirth, abortion, and heavy physical activities (11–13).

In Ethiopia, the proportion of newborns with LBW has been reported to be between 8 and 28% (14, 15). A Demographic and Health Survey (DHS) in 2016 suggested a national level of 13% (16). A national survey published in 2019 estimated that 48% of births took place in health facilities (17). This means that more than half of Ethiopian births are non-institutional, and data on them, including birth weights, are not routinely recorded.

An earlier study in Ethiopia showed a 37% increased risk of LBW in pregnancies exposed to IPV; however, this study was only undertaken at two hospitals (18). Women exposed to IPV may prefer to deliver at home (19). We recently reported the prevalence of IPV among pregnant women in Ethiopia to be around 20%, with a higher prevalence among rural women than among urban women (20). Therefore, the aim of the current study was to investigate the association between exposure to IPV during pregnancy and birth weight within a community involving women in different home settings.

## Materials and methods

### Study setting

The study was conducted in the Wondo Genet district located in the former Sidama zone of the Southern Nations, Nationalities, and Peoples Region (SNNPR). Based on the 2007 census and an annual population growth rate of 2.7% (21), the district's projected total population in 2017 was 200 078. The number of women of reproductive age was estimated to be 46 618 with 6 923 expected pregnancies in a year. Wondo Genet district has a high population density and ethnic diversity. The district has three urban and 12 rural kebeles (Kebele is the smallest administrative unit in Ethiopia). There are 16 health posts and five health centers serving the population. The nearest hospital is in a neighboring district. Among the pregnant women attending antenatal care, 88% had four visits in 2016; only 10% of women in this area delivered at home (Wondo Genet district health office report, 2017). The 2016 Ethiopian DHS indicated that around 69% of women attended antenatal care at least once during their last pregnancy and institutional delivery was 26% in the region (SNNPR). Among women who delivered their most recent live birth in a health facility, 53% of them stayed in the facility for up to 11 h following vaginal birth (16). In this study area, women are often discharged from health facilities 6 h after a normal delivery.

### Study design and participants

This was a community-based prospective cohort study investigating IPV. It was conducted between February and December 2017 among pregnant women who were enrolled at gestational age 25–34 weeks as listed by health extension workers (22) in two urban and three rural kebeles of the Wondo Genet district. The mothers and their babies were visited at home. We excluded twins and mother–baby pairs with late (i.e., invalid) birth weight measurements. In this article, we focused on IPV and its association with birth weight.

### Sample size and sampling procedure

The sample size to investigate the association between IPV and birth weight was calculated using OpenEpi version 3.03 software (23). The total sample size was estimated to be at least 435 based on an average birth weight of 3,000 g among unexposed and 2,850 g among exposed, with standard deviations of 423 g and 450 g, a ratio of unexposed to exposed of 4:1 (20), 80% statistical power, and 95% confidence level. The present work was part of a larger project (20) that required a sample size larger than this, so the sample size requirement for this study was met.

Pregnant women living in the selected kebeles were enrolled through home visits. The sites were identified as being “urban” or “rural” according to the Ethiopian DHS definition of these terms (16). The selection of kebeles was decided based on the number of pregnant women in the areas as reported by the health extension workers. Pregnant women who fulfilled the inclusion criteria were consecutively enrolled in the study until the required sample size was obtained.

### Variables

The main outcome variable was birth weight measured in grams. The main exposure variable for this study was IPV during pregnancy, assessed in a home visit at enrollment using questions adapted from the WHO multi-country study questionnaire on women's health and domestic violence against women (24). In the present study, IPV exposure in “the past 12 months” in the WHO study was changed to “during this pregnancy.” IPV was classified as being physical, sexual, or emotional. The respondents were given examples of physical violence including: partner had slapped or thrown something at her that could hurt her, pushed or shoved her, hit her with fist or something else that could hurt her, kicked, dragged or beaten her up, choked or burnt her on purpose, threatened to use or actually used a gun, knife, or other weapon against her. Examples of sexual violence included: partner had physically forced her to have sexual intercourse when she did not want to, had sexual intercourse when she did not want to because she was afraid of what her partner might do, and had forced her to do something sexual that she found degrading or humiliating. Examples of emotional violence included: partner had insulted her or made her feel bad about herself, had belittled or humiliated her in front of other people, had done things to scare or intimidate her on purpose, and had threatened to hurt someone she cared about. If the woman had experienced any of the three types of violence defined above, she was categorized as “IPV exposed.”

Other covariates included the mother's age (years), educational status of the mother and her partner (no education/primary/secondary and above), monthly income (Ethiopian Birr), residence (rural/urban), prior history of preterm birth and stillbirth (no/yes), antenatal care at least one visit (no/yes), smoking (no/yes), and regular alcohol use (no/yes) by participants or their partner. The Edinburgh Postnatal Depression Scale (EPDS) was used to measure maternal depression (25), and has been validated by previous studies in Ethiopia (26). Each of the 10 items in the EPDS has scores of 0–3; giving a maximum score of 30. Maternal depression was measured as a continuous variable with an EPDS score; we defined depression in this analysis as an EPDS score of 13 or more (27). A Maternity Social Support Scale with six items was used to measure social support. Each item has a score of 1–5, and the total score ranges from 6 to 30. Social support was categorized as low (score 0–18), medium (score 19–24), and high (score > 24) (28). Maternal malnutrition was assessed by mid-upper arm circumference (MUAC) and was measured in centimeters; undernutrition was set at MUAC <23 cm. The sex of the newborn (male/female/unknown) and its birth order (first/second and above) were recorded.

### Data collection

The data were collected between February and December 2017. The data used to achieve the objectives of the present study are the same as described for the previously mentioned survey of IPV in the study area (20), with the same baseline data. We added follow-up data on birth characteristics, such as time of birth; whether the birth was live or not; sex of the newborn, and birth weight collected at a home visit as soon as possible within 72 h (time after delivery was noted for each participant). We collected data on selected variables that could be potential confounders. The main exposure variables were collected by trained data collectors using a structured and pretested questionnaire. The exposure status was determined based on the survey data from the baseline study. Birth weight was measured using a digital baby scale (Beurer BY 80), and the reliability of the scale was routinely checked by regularly measuring something of known weight. The mid-upper arm circumference (MUAC) was measured using a centimeter tape at the midpoint between the shoulder and elbow with the arm hanging down at the side relaxed. A MUAC below 23 cm defined a participant as being “malnourished.” The woman was asked for the date of her last menstrual period, which was used to calculate gestational age. When the exact date was unknown, the mother was asked to provide the alleged month.

The field assistants actively sought out the women in person to check for delivery. They also used mobile phones. In addition, they were notified through mobile phone by the 1-to-5 network leaders as well as by the participants themselves, so that they recorded the exact date and time of birth for all births, whether they took place at home or at a health facility. The field assistants measured birth weight using a digital scale as soon as possible after delivery according to the operating instructions. They also recorded whether it was a live birth or stillbirth. Based on the last menstrual period, the principal investigator later determined whether the birth was term or not.

### Data analysis

Data were analyzed using SPSS version 20 (Armonk, NY: IBM Corp. USA) software. Chi-square and Fisher's exact tests were used to compare categorical variables. Mean values were compared using *t*-tests and analyses of variance. Multiple linear regression was performed to investigate the association between birth weight and maternal exposure to IPV during pregnancy (shown in **Table 3**). Other selected determinants were also studied. Preliminary analyses ensured that there was no violation of the assumption of normality, linearity, multicollinearity, and homoscedasticity. Complete case analysis was used for missing values to minimize potential bias. Variables having a correlation with birth weight at *p* < 0.2 levels and socio-economic and demographic variables were entered in the adjusted regression model. Maternal age, MUAC, and monthly income were entered as continuous variables. An adjusted regression coefficient (B) with a 95% confidence interval was reported. A *p*-value was considered statistically significant when < 0.05. Sensitivity analysis was also performed in a group of neonates whose birth weights were taken within the first 48 h after birth. We also analyzed risk factors for LBW (birth weight < 2,500 g) using logistic regression analysis (data not presented in a table).

### Ethical consideration

The study was conducted after obtaining approval from the Institutional Review Board (IRB) at the College of Medicine and Health Sciences, Hawassa University (Ref No: IRB/006/09) and regional ethical committee of Western Norway (Ref No: 2016/1908/REK vest). Permission from the parents as well as assent was obtained for those <18 years. In this setting, written consent was not culturally acceptable, but participants were comfortable with oral consent. Informed oral consent was obtained from each participant >18 years and recorded by the interviewer, according to the protocol approved by IRB. The study followed the ethical and safety guidelines recommended by the World Health Organization (29). All women who participated in the study were given information about the psychological and legal support available and how access could be provided if needed. This support would be paid for by the study project.

## Results

Out of 589 pregnant women enrolled in the large follow-up study, we excluded 84 cases from the analysis: including eight women with twins, one preterm and two stillborns, 22 cases where the mother refused to have the baby weighed, 26 assessed after 72 h, two early neonatal deaths and 23 lost to follow-ups.

Table 1a shows the socio-demographic characteristics of the pregnant women enrolled in the large follow-up study, comparing women whose babies were weighed with those whose babies were not. Women with missing birth weights formed a significantly lower proportion of these participants. They tended to be rural dwellers, women with no formal education, women of the protestant religion, and women whose husbands were farmers. Otherwise, the characteristics of these women were not significantly different from the women for whom birth weights were collected. Among the 505 mother–singleton newborn pairs who were analyzed for birth weight, the mean age of the mothers was 25 years, ranging from 16 to 45. A quarter (26%) of the women had no formal education and many (80.8%) were housewives. Only five participants (1.0%) responded that during the current pregnancy they had been drinking alcohol and one (0.2%) that she had been smoking; therefore, alcohol and smoking were not included in any further analysis of determinants. Among the 505 births, 365 (72.3%) took place at health facilities and 140 (27.7%) were home deliveries.

**Table 1A:** Socio-demographic characteristics of pregnant women enrolled in the study whose baby's birth weight was measured or not, in Wondo Genet district, Ethiopia, 2017.

**Characteristics**	**Birth weight measurement**			
	**Total participants, *n* (%)**	**Missing,** ***n* (%)**	**Measured, *n* (%)**	***P*** **-value**
Total	589 (100)	84 (100)	505 (100)	
**Residence**				
Rural	304 (51.6)	32 (38.1)	272 (53.9)	0.007
Urban	285 (48.4)	52 (61.9)	233 (46.1)	
**Age of the mother**				
15–24 years	233 (39.6)	35 (41.7)	198 (39.2)	0.670
25–45 years	356 (60.4)	49 (58.3)	307 (60.8)	
**Education of the mother**				
No education	145 (24.6)	12 (14.3)	133 (26.3)	0.018
Formal education	444 (75.4)	72 (85.7)	372 (73.7)	
**Occupation of the mother**				
Housewife	472 (80.1)	64 (76.2)	408 (80.8)	0.328
Others	117 (19.9)	20 (23.8)	97 (19.2)	
Monthly income (ETB^*^)				
<1,500	273 (46.3)	38 (45.2)	235 (46.5)	0.742
1,500–2,999	214 (36.3)	29 (34.5)	185 (36.6)	
≥3,000	102 (17.3)	17 (20.2)	85 (16.8)	
**Religion of the mother**				
Protestant	540 (91.7)	71 (84.5)	469 (92.9)	0.010
Others	49 (8.3)	13 (15.5)	36 (7.1)	
**Alcohol drinking by mother**				
No	583 (99.0)	83 (98.8)	500 (99.0)	1.000
Yes	6 (1.0)	1 (1.2)	5 (1.0)	
**Husband's education**				
No education	113 (19.2)	11 (13.1)	102 (20.2)	0.091
Primary	188 (31.9)	23 (27.4)	165 (32.7)	
Secondary and above	288 (48.9)	50 (59.5)	238 (47.1)	
**Husband's occupation**				
Farmer	251 (42.6)	23 (27.4)	228 (45.1)	0.011
Merchant	168 (28.5)	26 (31.0)	142 (28.1)	
Government employee	44 (7.5)	9 (10.7)	35 (6.9)	
Others	126 (21.4)	26 (31.0)	100 (19.8)	
**Alcohol drinking by husband**				
No	527 (89.5)	74 (88.1)	453 (89.7)	0.657
Yes	62 (10.5)	10 (11.9)	52 (10.3)	
**Social support**				
Low	40 (6.8)	9 (10.7)	31 (6.1)	0.247
Medium	126 (21.4)	15 (17.9)	111 (22.0)	
Adequate	423 (71.8)	60 (71.4)	363 (71.9)	

* ETB, Ethiopian Birr. P-value from chi-square test.

Table 1b shows the clinical characteristics of the pregnant women enrolled in the large follow-up study, comparing women whose babies were weighed with those whose babies were not, and there was no significant difference between them. The mean maternal MUAC was 24.2 (standard deviation = 1.9) cm.

**Table 1b:** Clinical characteristics of pregnant women enrolled in the study whose baby's birth weight was measured or not, in Wondo Genet district, Ethiopia, 2017.

**Characteristics**	**Birth weight measurement**			
	**Total participants, *n* (%)**	**Missing,** ***n* (%)**	**Measured, *n* (%)**	***P*** **-value**
Total	589 (100)	84 (100)	505 (100)	
MUAC^*^				
>23 cm	504 (85.6)	75 (89.3)	429 (85.0)	0.295
<23 cm	85 (14.4)	9 (10.7)	76 (15.0)	
IPV^*^exposure				
Not exposed	464 (78.8)	69 (82.1)	395 (78.2)	0.415
Exposed	125 (21.2)	15 (17.9)	110 (21.8)	
**Depression**				
No	549 (93.2)	76 (90.5)	473 (93.7)	0.282
Yes	40 (6.8)	8 (9.5)	32 (6.3)	
**Prior stillbirth**				
No	555 (94.2)	80 (95.2)	475 (94.1)	0.805
Yes	34 (5.8)	4 (4.8)	30 (5.9)	
**Prior preterm birth**				
No	558 (94.7)	81 (96.4)	477 (94.5)	0.602
Yes	31 (5.3)	3 (3.6)	28 (5.5)	
**Birth order**				
First	129 (21.9)	23 (27.4)	106 (21.0)	0.190
Second and above	460 (78.1)	61 (72.6)	399 (79.0)	
ANC^*^				
No	140 (23.8)	17 (20.2)	123 (24.4)	0.412
Yes	449 (76.2)	67 (79.8)	382 (75.6)	
**Delivery place**				
Home	163 (28.8)	23 (37.7)	140 (27.7)	0.104
Health facility	403 (71.2)	38 (62.3)	365 (72.3)	
**Sex of the baby** ^**^#^**^				
Male	304 (54.1)	29 (50.9)	275 (54.5)	0.607
Female	258 (45.9)	28 (49.1)	230 (45.5)	

* MUAC, mid-upper arm circumference; IPV, intimate partner violence; ANC, antenatal care.

# Sex of the baby does not add up 84 in the missing birth weight group due to lost to follow-up, early neonatal deaths, and stillbirths. P-value from the chi-square test.

Birth weight was assessed within 24 h for 234 (46.3%) newborns, between 24 and 48 h for 243 (48.1%) newborns, and between 48 and 72 h for 28 (5.5%) newborns. Mean birth weight by mother's characteristics is shown in Tables 2a,b. The mean birth weight among all singleton newborns was 3,222 grams (range 2,048–4,325 g) and was similar irrespective of the day of measurement: Day 1 mean 3,205 g (95% CI 3,147–3,264 g); Day 2 mean 3,244 g (95% CI 3,183–3,305 g); and Day 3 mean 3,159 g (95% CI 2,946–3,372 g). The mean birth weight among newborns of women who had experienced IPV was lower (3,054 g, 95% CI 2,962–3,147) than among newborns of women not exposed to IPV (3,269 g, 95% CI 3,223–3,314). The mean birth weight was significantly higher among newborns of Protestant mothers; mothers whose husbands did not drink alcohol; and mothers with no prior history of preterm birth and stillbirth. In a one-way ANOVA, there was no statistically significant difference in the mean birth weight between the different categories of residence, age, occupational status, MUAC, birth order, and sex of the newborn, and also among the different categories of income, educational status of the woman, educational status, and occupational status of the husband (Tables 2a,b).

**Table 2a:** Mean birth weight of babies by socio-demographic characteristics of their mothers in Wondo Genet district, Ethiopia, 2017.

**Variable**	**Birth weight**			
	***N*** **(%)**	**Mean (gram)**	(95% CI^*^)	***p*** **-value**
**Total**	505 (100)	3,222	(3,181–3,263)	
**Residence**				
Rural	272 (53.9)	3,237	(3,181–3,292)	0.454
Urban	233 (46.1)	3,205	(3,142–3,267)	
**Age of the mother**				
15–24	198 (39.2)	3,223	(3,157–3,289)	0.962
25–45	307 (60.8)	3,221	(3,168–3,274)	
**Education of the mother**				
No education	133 (26.3)	3,173	(3,086–3,260)	0.356
Primary	244 (48.3)	3,233	(3,173–3,293)	
Secondary and above	128 (25.3)	3,252	(3,176–3,328)	
**Occupation of the mother**				
Housewife	408 (80.8)	3,231	(3,183–3,278)	0.345
Others	97 (19.2)	3,185	(3,102–3,268)	
Monthly income (ETB^*^)				
<1,500	235	3,254	(3,196–3,313)	0.274
1,500–2,999	185	3,207	(3,138–3,277)	
≥3000	85	3,163	(3,056 −3,270)	
**Religion of the mother**				
Protestant	469 (92.9)	3,235	(3,192–3,277)	0.028
Others	36 (7.1)	3,055	(2,896–3,215)	
**Alcohol drinking by the mother**				
No	500 (99.0)	3,224	(3,183–3,266)	0.252
Yes	5 (1.0)	2,981	(2,370–3,592)	
**Husband's education**				
No education	102 (20.2)	3,190	(3,096–3,284)	0.746
Primary	165 (32.7)	3,234	(3,160–3,308)	
Secondary and above	237 (46.9)	3,226	(3,166–3,285)	
**Husband's occupation**				
Farmer	228 (45.1)	3,219	(3,158–3,279)	0.443
Merchant	142 (28.1)	3,257	(3,175–3,340)	
Government employee	35 (6.9)	3,267	(3,097–3,436)	
Others	100 (19.8)	3,163	(3,074–3,252)	
**Alcohol drinking by husband**				
No	453 (89.7)	3,240	(3,197–3,283)	0.012
Yes	52 (10.3)	3,066	(2,924–3,208)	
**Social support**				
Low	31 (6.1)	3,300	(3,183–3,416)	0.335
Medium	111 (22.0)	3,172	(3,078–3,267)	
High	363 (71.9)	3,231	(3,182–3,279)	

* CI, confidence interval; ETB, Ethiopian Birr. P-values were obtained from t-test and one-way ANOVA.

**Table 2b:** Mean birth weight of babies by clinical characteristics of their mothers in Wondo Genet district, Ethiopia, 2017.

**Variable**	**Birth weight**			
	***N*** **(%)**	**Mean (gram)**	(95% CI^*^)	***p*** **-value**
**Total**	505 (100)	3,222	(3,181–3,263)	
MUAC^*^				
≥23 cm	429 (85.0)	3,224	(3,180–3,269)	0.791
<23 cm	76 (15.0)	3,209	(3,094–3,323)	
IPV^*^exposure				
Not exposed	395 (78.2)	3,269	(3,223–3,314)	<0.001
Exposed	110 (21.8)	3,054	(2,962–3,147)	
**Depression**				
No	473 (93.7)	3,217	(3,174–3,261)	0.413
Yes	32 (6.3)	3,288	(3,142–3,435)	
**Prior stillbirth**				
No	475 (94.1)	3,398	(3,260–3,536)	0.035
Yes	30 (5.9)	3,211	(3,168–3,254)	
**Prior preterm birth**				
No	477 (94.5)	3,418	(3,272–3,565)	0.023
Yes	28 (5.5)	3,210	(3,168–3,253)	
ANC^*^				
No	123 (24.4)	3,210	(3,126–3,293)	0.739
Yes	382 (75.6)	3,226	(3,178–3,274)	
**Delivery place**				
Home	140 (27.7)	2,953	(2,881–3,026)	<0.001
Health facility	365 (72.3)	3,325	(3,279–3,371)	
**Birth order**				
First	106 (21.0)	3,268	(3,183–3,352)	0.263
Second and above	399 (79.0)	3,210	(3,162–3,257)	
**Sex of baby**				
Male	275 (54.5)	3,255	(3,197–3,312)	0.090
Female	230 (45.6)	3,183	(3,124–3,242)	

* CI, confidence interval; MUAC, mid-upper arm circumference; IPV, intimate partner violence; ANC, antenatal care. P-values were obtained from t-test and one-way ANOVA.

Associations between birth weight and potential determinants including maternal exposure to IPV during pregnancy were analyzed by multivariable linear regression and are shown in Table 3. In adjusted regression analysis babies of mothers exposed to IPV had 203 g (B −203 95% CI −320 to −87) lower birth weight than babies of mothers without. Babies born at health facilities had higher birth weight than those born at home; girls had a lower birth weight than boys; and increasing gestation was associated with higher birth weight. Prior stillbirth and prior preterm delivery were associated with birth weight in the crude analysis but were not statistically significant in the adjusted analysis.

**Table 3:** Multivariable linear regression of factors associated with birth weight in Wondo Genet district, southern Ethiopia, 2017 (*n* = 505).

**Variables**	**Crude B**	95% CI^*^	**Adjusted B**	95% CI^*^
IPV exposure^*^	−215	−313 to −116	−203	−320 to −87
Age in years (increase by 1 year)	2.4	−7 to 12	4	−7 to 16
Any maternal education	67	−27 to 160	71	−18 to 160
Residence (urban compared to rural)	−32	−115 to 51	3	−79 to 85
House wife compared to other maternal occupation	46	−59 to 151	85	−11 to 182
Protestant compared to other religion	179	19 to 339	104	−39 to 247
Alcohol drinking by husband	−174	−309 to −39	−49	−185 to 86
Monthly income (increase by 100 ETB^*^)	−3	−7 to 1	−4	−8 to 0
Social support (increase by total score)	7	−3 to 17	3	−10 to 16
Maternal depression (increase by total score)	1	−8 to 10	2	−9 to 13
Parity (increase in number)	−3	−26 to 21	5	−26 to 35
Maternal MUAC (increase by 1 cm)	−10	−32 to 12	7	−13 to 28
Prior history of preterm birth	208	28 to 388	106	−63 to 276
Prior history of still birth	187	13 to 362	143	−21 to 307
Antenatal care use	16	−80 to 113	−25	−111 to 62
Facility delivery compared to home delivery	372	286 to 458	340	256 to 424
Female baby compared to male	−72	−154 to 11	−131	−206 to −57
Increase in 1 week of gestation	86	64 to 109	79	58 to 101

* CI, confidence interval; ETB, Ethiopian Birr; IPV, intimate partner violence; B is unstandardized beta coefficient.

Regression coefficients were obtained from multivariable regression analysis. Monthly income and week of gestation at birth, MUAC, social support, depression, parity, and age were entered as continuous variables. The value of the regression coefficient is for yes, i.e., yes is compared to no for IPV exposure, any maternal education, alcohol drinking by husband, preterm birth, stillbirth, and antenatal care use. Model was considered significant at p < 0.001, R^2^ of the model = 0.283. IPV was adjusted for: all variables in the table.

Low birth weight (<2,500 g) was found in 42 babies (8.3%). It was identified among 23 of the newborns of women exposed to IPV (20.9%; 95% CI 14.1–29.3), compared to 19 among newborns of women not exposed to IPV (4.8%; 95% CI: 3.0–7.3). This represents a risk ratio of 4.3.

Maternal undernutrition (thinness) and exposure to IPV during pregnancy were significantly associated with LBW in the multivariate logistic regression analysis after controlling for potential confounders. LBW was eight times more likely to occur in women exposed to IPV during pregnancy (AOR: 7.8; 95% CI: 3.4–17.7) and five times more likely in women with undernutrition (thinness) (AOR: 5.4; 95% CI: 2.0–14.1). Another factor associated with LBW was place of delivery, with around five times increased likelihood of LBW in home deliveries (AOR: 5.4; 95% CI: 2.5–11.6) than in facility deliveries, both crude and adjusted. The result did not change when analyzing the effects of interaction between IPV and nutrition (not shown in Tables).

## Discussion

In this community-based follow-up study of 505 mother–singleton infant pairs in southern Ethiopia, the babies from IPV-exposed pregnancies were on average almost 200 g smaller. Among the women who experienced IPV during pregnancy, one in five gave birth to babies with low birth weight, whereas one in 20 unexposed women gave birth to babies with low birth weight.

Associations between IPV and low birth weight have been reported by studies conducted in Egypt (30), Vietnam (31), and also in Ethiopia in Harari (18) and Tigray (32) regions. The large hospital-based Egyptian study found that pregnant women who experienced IPV had a double risk of LBW compared with women who had not (30). The Vietnamese study reported that women who were exposed to physical violence were six times more likely to have LBW infants (31). A potential mechanism for IPV causing low birth weight is a direct mechanism through trauma, perhaps causing damage to the placenta resulting in effects on the fetal nutrient supplies, premature rupture of membranes, premature labor, and low birth weight (33). It may also involve an indirect mechanism *via* related risk factors, such as the use of alcohol, resulting in impaired fetal growth (34). Other factors include changes in nutritional habits or reduced food intake, perhaps as a result of loss of appetite during depression (35). Moreover, a Norwegian nationwide population-based prospective cohort study also did not find any significant association between violence during pregnancy and low birth weight (36). In the Norwegian study, the significant crude association between sexual violence and LBW became no longer significant when adjusted for socio-demographic factors. But we found no change in the adjusted regression coefficient (B) when we controlled for socio-economic and demographic variables in our analysis. The authors also acknowledged that the lack of association could be related to the low prevalence of abuse noted during the pregnancy, as they assessed only sexual IPV once at a gestational week of 17 and may have missed any violence that occurred later.

We observed lower birth weight among girls than boys and this finding has been observed in many other studies and reports (37). Female baby becomes highly significant in the adjusted model while not significant in the crude analysis. It is certainly a known fact that female babies weigh less than boys. There may be confounding here if more girls of low gestational age were counted. Adjusting for gestational age in the model made the difference statistically significant. When adjusted for socio-economic status, we found that birth weight was over 300 g higher at a health facility than at home. Similar results had been reported from Nepal (38), Bangladesh (39), and India (11). This could partly reflect that IPV-exposed women are more likely to deliver at home. In addition, premature labor is more likely to happen at home since delivery is not yet expected, and hence smaller babies from premature births are more likely. In our study, mothers' socio-economic status and partners' alcohol consumption did not affect birth weight. It may be because of the setting where most participants are poor and few are rich. This reduces the statistical power of our analysis. Alcohol drinking was a strong predictor in the crude analysis but adjusting by IPV reduced the effect. Surprisingly, there was only one preterm birth. The pooled prevalence of preterm birth in Ethiopia was 10.5% in a systematic review (40), a community-based study from rural Ethiopia that assessed intrauterine growth patterns using ultrasound measurement found a 4.9% preterm birth (41), and a study in Hawassa town found a 3.6% of preterm birth (42). The lower prevalence of preterm birth in this study might be due to a recall bias of the last menstrual period.

In line with our figure of LBW (8.3%), the study that was conducted in rural Ethiopia reported a LBW of 7.9% (41). But, it was not as common in our study as reported by the Ethiopian DHS, 2016 (13%) (16). However, we noticed that the DHS data were based on birth weight collected from both written records and subjective assessment reports by the mother from births that occurred within 5 years of the survey, which could be imprecise and subject to recall bias. Also, the lower prevalence of low birth weight in our study may be partly due to the nutrition screening program that was transformed from being campaign-based into routine activities in the region, and the scaling up of nutrition-specific interventions in the country. However, this program had just started when our data collection was done and likely had little impact on our study (43). Another possibility is that the women lost to follow-up could have a higher proportion of small babies, but we have no way of confirming this. Women who had babies with no recorded birth weight were fairly similar to the rest in terms of their other baseline characteristics, and their effect of exclusion on the main result is likely small.

This study had several strengths. A reasonably large study population of pregnant women was enrolled consecutively in the area, and the participants were, as far as we know, representative for the area. This ensured a good representation of rural women and home births.

The study also had some limitations. A few pregnant women may have been missed in the households, including women who try to hide their pregnancy, and we have no information about this. The birth weight was missing for 14% of the women including 4% who were lost to follow-up. However, there were no large differences in their background characteristics, and this likely represented no major selection bias. The proportion of LBW was slightly higher (8.3%) among those measured at 0–72 h than when measurements were limited to 0–48 h (7.7%), but this had little impact on the association between IPV and birth weight in our sensitivity analysis (see Supplementary Tables 1, 2). An early ultrasound before 24 weeks of gestation is important for accurate gestational age estimation (44) but this was not possible and we calculated the gestational age using the woman's reported date of last menstrual period, which may also be subject to recall bias. Furthermore, some variables that could affect birth weight like medical diseases, body mass index, weight gain during pregnancy, and prior history of LBW were not collected and some residual confounding effects cannot be excluded.

## Conclusion

Women exposed to intimate partner violence during pregnancy tended to have smaller babies than unexposed women. Birth weight was also associated with the sex of the newborn, gestational age at birth, and place of delivery. Antenatal clinics may consider routinely identifying women suffering from IPV and then referring them to the relevant organizations. The positive efforts by the Ministry to enhance facility delivery should include identifying babies with lower birth weights who need special care.

## Data availability statement

The original contributions presented in the study are included in the article/supplementary files, further inquiries can be directed to the corresponding author/s.

## Ethics statement

The studies involving human participants were reviewed and approved by Institutional Review Board (IRB) at the College of Medicine and Health Sciences, Hawassa University (Ref No: IRB/006/09) and regional Ethical Committee of Western Norway (Ref No: 2016/1908/REK vest). Written informed consent to participate in this study was provided by the participants' legal guardian/next of kin. Informed oral consent was obtained from each participant > 18 years and recorded by the interviewer.

## Author contributions

SB conceptualized, designed and implemented the study, analyzed the data, and drafted the manuscript. AA and SGH contributed with planning, analyzed the data, revised and edited the manuscript, and supervised. All authors have read and approved the final manuscript.

## Funding

This research was funded by the NORHED/SENUPH (Norwegian Program for Capacity Development in Higher Education and Research for Development/South Ethiopia Network of Universities in Public Health), Agreement no. ETH13/0025. The funder had no role in study design, data collection and analysis, decision to publish, or preparation of the manuscript.

## Conflict of interest

The authors declare that the research was conducted in the absence of any commercial or financial relationships that could be construed as a potential conflict of interest.

## Publisher's note

All claims expressed in this article are solely those of the authors and do not necessarily represent those of their affiliated organizations, or those of the publisher, the editors and the reviewers. Any product that may be evaluated in this article, or claim that may be made by its manufacturer, is not guaranteed or endorsed by the publisher.
