# Supplementary material for: Birth weight was associated with maternal exposure to intimate partner violence during pregnancy in southern Ethiopia: A prospective cohort study
Source: Front Public Health. 2022 Nov 2;10:960443. doi: 10.3389/fpubh.2022.960443 (PMC9667023; doi:10.3389/fpubh.2022.960443)
Supplement: Supplementary file 1 [file Data_Sheet_1.doc]

**Table S1**

Sensitivity analysis on simple linear regression of the association of IPV with birth weight, comparing birth weight taken on days 1 and 2 with birth weights taken on days 1, 2 and 3 in Wondo Genet district, Southern Ethiopia, 2017

| **Variables** | **Crude B** | **95% CI** | **p-value** |
| --- | --- | --- | --- |
| **IPV exposure (yes)** |  |  |  |
| Birth weight taken on day 1 and 2 (n=477) | -219 | -318, -119 | 0.000 |
| Birth weight taken on day 1, 2 and 3 (n=505) | -215 | -313, -116 | 0.000 |

Note: The adjustment was done to see whether or not the result is affected, if we analyse only birth weight taken on days 1 and 2 and to compare with birth weight taken on days 1, 2 and 3.

**Table S2**

Sensitivity analysis on simple linear regression of the association of IPV with birth weight, comparing weight loss adjustment with a total sample size of 505 in Wondo Genet district, Southern Ethiopia, 2017

| **Variables** | **Crude B** | **95% CI** | **p-value** |
| --- | --- | --- | --- |
| **IPV exposure (yes)** |  |  |  |
| Birth weight adjusted for weight loss (n=505) | -197 | -294, -99 | 0.000 |
| Original birth weight (n=505) | -215 | -313, -116 | 0.000 |

Note: We do not have weight loss when we compare birth weight taken on days 1, 2 and 3. The adjustment was done to see whether or not the result is affected, if there was weight loss on days 2 and 3 by subtracting 5% of the birth weight measurement from day 2 and 10% from day 3 and to compare with the original.
